# Integrated Evaluation of Coupling Coordination for Land Use Change and Ecological Security: A Case Study in Wuhan City of Hubei Province, China

**DOI:** 10.3390/ijerph14111435

**Published:** 2017-11-22

**Authors:** Ji Chai, Zhanqi Wang, Hongwei Zhang

**Affiliations:** Department of Land Resource Management, School of Public Administration, China University of Geosciences (Wuhan), 388 Lumo Road, Hongshan District, Wuhan 430074, China; chaiji_cug@163.com (J.C.); zhangfocus@cug.edu.cn (H.Z.)

**Keywords:** ecological security, land use change, emergy analysis, coupling coordination degree model, Wuhan city

## Abstract

As land resources and ecosystems provide necessary materials and conditions for human development, land use change and ecological security play increasingly important roles in sustainable development. This study aims to reveal the mutual-influence and interaction between land use change and ecological security in Wuhan, based on the coupling coordination degree model. As such, it provides strategies for the achievement of the synchronous and coordinated development of urbanization and ecological security. The results showed that, during the period from 2006 to 2012, the size of built-up area in Wuhan increased to 26.16%, and that all the other types of land use reduced due to the urbanization process, which appeared to be the main driving force of land use change. The ecological security in Wuhan has been improving as a whole although it was somewhat held back from 2006 to 2008 due to the rapid growth of built-up area. The coupling coordination analysis revealed that the relationship between built-up area and ecological security was more coordinated after 2008. The results can provide feasible recommendations for land use management and environmental protection from the viewpoint of coordinated development. To achieve sustainable development from economic and ecological perspective, policy makers should control the rate of urban expansion and exert more effort on intensive land use, clean energy development and emission reduction.

## 1. Introduction

Ecological security, as the fundamental guarantee for achieving the sustainable development and promoting public health, has already become a widely used security concept and important research topic because of serious ecological degradation and environmental pollution [1,2]. There is ample evidence that ecosystems in many regions have become highly stressed and dysfunctional for various reasons [3]. Ecological security can be affected by natural factors, such as global climate change and natural disasters [4]. In addition, human activities can also influence the ecological security through consuming natural resources and discharging waste while they are developing economy and creating wealth [5]. Among these factors, land use change, as the most common human activity, plays an essential role in ecological security. Land use change can directly transform landscapes and affect the biodiversity and eco-productivity, which may increase ecological risks [6]. What is more, with rapid urbanization processes, it can cause serious environmental pollution and threaten ecological security and public health.

Land resource is the physical basis that supports the existence and development of human beings [7,8]. Land use change is a manifestation of the mutual-influence and interaction between human beings and nature [9]. Accompanying the rapid development of economy and urbanization, great changes have taken place in land use. The demand for infrastructure for housing, businesses, and industry caused by the expanding built-up area is often satisfied through land use change to exploit natural lands such as cultivated land, forest, water bodies, and open spaces [10]. Except for such effects, land use change, especially the increase of urban construction, can seriously influence the ecosystem by producing various pollutants, which can destroy the environment including the climate, soil, and biotic components. Due to excessive exploitation and utilization of land resources, the ecological environment in many places has been damaged, gravely threatening the ecological security [11]. Meanwhile, as people pay more and more attention to eco-environmental problems, governments have taken measures to protect the ecological environment and cut down on pollution, such as policies of holistic control over the construction land and ecological civilization campaign in China. Therefore, to strengthen ecological protection and realize rational land utilization, a comprehensive evaluation of land use change and ecological security is urgently needed.

Since the International Geosphere-Biosphere Program (IGBP) and the International Human Dimensions Program (IHDP) launched a related research project—the Global Change and Terrestrial Eco-systems Project (GCTE)—studies about land use and ecological environment have been carried out by scholars across the world [12]. Wessels K.J. et al. assessed the effects of land degradation in the former homelands of northern South Africa which was caused by human activities with a 1 km AVHRR NDVI time-series [13]. Xu L.Y. et al. used a comprehensive method, named Double Land Ecological Security, to evaluate the land ecological security in regional and unit scales for reasonable and specific urban planning in Guangzhou, China [14]. Gong J. et. al conducted a spatially explicit landscape ecological risk analysis and a simulation-driven scenario analysis in Ezhou City, China to explore for measures for the sustainable development of ecologically vulnerable land systems [15]. Li Y. et al. analyzed the future characteristics of land use change using the CLUE-S model and explored landscape ecological risk responses to land use change using the landscape ecological risk index method [16]. However, most of the previous research focused on evaluating the impacts of land use change on ecosystems in some regions, while the study of the interaction between land use change and ecological security and their coordinated development is still in its infancy. In this paper, we attempt to investigate interaction and level of compatibility between this two aspects by coupling coordination analysis in order to support more coordinated development of urbanized system. Moreover, due to the complexity of urban system, traditional methods generally selected many indices to comprehensively evaluate the state of the system. This had disadvantages such as overlapping information and inconsistent units of indicators. In order to overcome these deficiencies, a unifying measure of emergy was introduced in this study to evaluate the level of ecological security.

This paper selected Wuhan, the capital city of Hubei Province in China, as the study area. The city is located at the Yangtze Plain along the middle reaches of the Yangtze River, with tens of lakes inside. The objectives of this study were to (1) analyze the land use change of the study area from 2006 to 2012 through numerical analysis using the land use dynamic degree model; (2) establish an evaluation system of ecological security using the method of emergy analysis to build the emergy-based ecological security index; (3) attempt to reveal the interaction between land use change and ecological security via the coupling coordination degree model; (4) analyze and improve the understanding of land use change and eco-environment so that a sustainable strategy can be established to promote their coordinated development. Our research can provide an overview of the interactional relationship between ecological security and land use in Wuhan. The results can offer new ideas for land management and for drafting future eco-environmental policies.

## 2. Study Area and Data

### 2.1. Study Area

**Figure 1:**
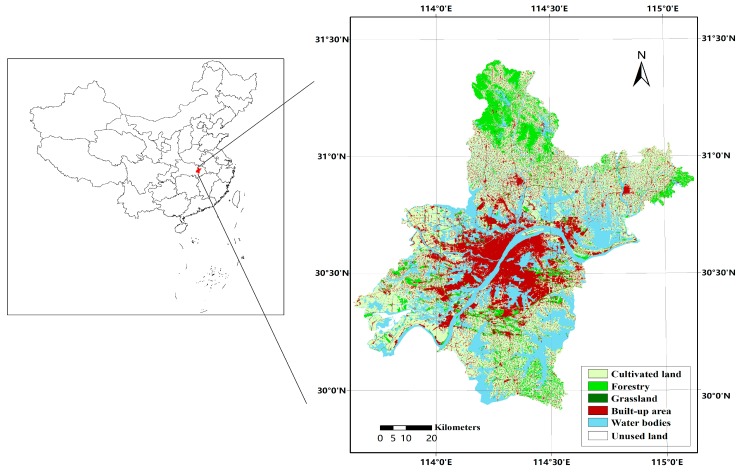
Location of the study area and its land use map of 2012.

Wuhan, the capital city of Hubei province, is one of the midland cities in China, located between 113°41′–115°05′ E and 29°58′–31°22′ N, covering 13 districts such as Jiangan district, Jianghan district, Hanyang district and Wuchang district, as shown in Figure 1. Wuhan lies in the east of The Jianghan Plain, in the middle of the Yangtze River, covering an area of over 8494 km^2^. The terrain of Wuhan is high in the east and lower in the west, most of which is less than 150 m above sea level. Moreover, Wuhan is one of the core cities of the Yangtze River Economic Zone, where the Yangtze River and Hanjiang River converge.

Since the reform and opening-up process in China, Wuhan has been developing fast in a comprehensive way. With abundant water resources, Wuhan is famous for rice cultivation and fishery. Wuhan’s privileged geographical location and good economic foundation have made it one of the developed cities in China with dynamic economic growth. In addition, water bodies accounts for nearly a quarter of the total area in Wuhan with nearly 100 lakes. Wuhan has been established as a national lake conservation region and ecologically representative region at the national level, playing an important role in the national ecological security. Demographically, the urban population in the study area first increased from 63.4% in 2006 to 65.0% in 2009, and then to 67.5% in 2012, according to the official data of the National Bureau of Statistics. Meanwhile, great changes have taken place regarding land use in Wuhan. According to the land use data, the total area of the cultivated land was 3612.44 km^2^ in 2006, which decreased by 219.02 km^2^ to 3393.42 km^2^ in 2012. In contrast, the built-up area increased greatly from 1304.75 km^2^ to 1646.11 km^2^ during the same period. The area of grassland declined slightly from 43.35 km^2^ to 35.99 km^2^, the forest land shrank from 1005.39 km^2^ to 980.14 km^2^, and the size of water bodies decreased from 2552.76 km^2^ to 2465.83 km^2^ during the same period. In terms of rapid economic development and land use change, Wuhan is a representative study area to explore the interaction between land use change and ecological security.

### 2.2. Data Sources

The land use data in this paper from 2006 to 2012 were provided by the Land Resources and Planning Bureau of Wuhan. The land use data were developed through remote sensing classification (from aerial imagery and SPOT 5 satellite imagery) and field validation (classification accuracy: 93–95%). The maps were land cover vector data generated by field investigation and human-computer-interaction interpretation. All of the land cover data were organized and re-projected into Universal Transverse Mercator projection. The dataset was based on the land use maps at a scale of 1:10,000. For the convenience of analysis, these maps were broken down to 6 classes including cultivated land, water bodies, built-up area, forestry, grassland and unused land based on land use status in accordance with the China land use/land cover remote sensing classification system by Liu J.Y. et al. [17].

The socio-economic data used in this paper were from Wuhan Statistical Year Book [18,19,20,21,22,23,24]. The data of pollutant emissions were from China Statistical Year Book on Environment [25,26,27,28,29,30,31]. The meteorological data used for emergy calculation were from China Meteorological Data Service Center [32].

## 3. Methods 

### 3.1. Analysis of Land Use Change

After the changes of land use structure and area during the study period were evaluated on the Arc INFO platform (ERSI, Redlands, CA, USA), the land use change was analyzed from the aspect of land use dynamic degree and land use integrated dynamic degree based on the findings of Wang et al. [33].

The land use dynamic degree is the rate of change of a certain type of land area during a period of time [34], which is used to describe the dynamic change of land use. The formula is described as

(1) $$R_{d}=\frac{X_{t_{2}}-X_{t_{1}}}{X_{t_{1}}}\times \frac{1}{t_{2}-t_{1}}\times 100\%$$

where *R_d_* refers to the land use dynamic degree, $X_{t_{2}}$ and $X_{t_{1}}$ represent the areas of a specific land use type at the beginning and end of the study, and *t*_1_ refers to the start of study period, *t*_2_ refers to the end of study period.

Land use integrated dynamic degree is the total land use change in the study area, which can quantitatively describe the rate of land use change [10]. The formula is as follows:

(2) $$R_{t}=\frac{\sum_{i=1}^{n}\left|X_{\left(i,t_{2}\right)}-X_{\left(i,t_{1}\right)}\right|}{\sum_{i=1}^{n}X_{\left(i,t_{1}\right)}}\times \frac{1}{t_{2}-t_{1}}\times 100\%$$

where *R_t_* refers to the land use integrated dynamic degree, $X_{\left(i,t_{1}\right)}$ represents the area of land use type *i* at the beginning of the study, $X_{\left(i,t_{2}\right)}$ represents the area of land use type *i* at the end of the study, and *t*_2_ − *t*_1_ refers to the study period.

### 3.2. Assessment of Ecological Security 

#### 3.2.1. Emergy Analysis

The emergence of the concept of emergy [35] provided us a new way to investigate ecosystems. According to the theory of emergy, emergy is defined as the sum of all flows of energy directly or indirectly required to provide a given product. The comparison of otherwise different forms of energy and therefore their comparison is made possible when all flows are expressed in one unit—solar emergy joules (seJ) [36]. To transform different kind of energy flows into emergy, unit emergy value (UEV) are used. UEV is defined as emergy (seJ) needed to provide one unit of the flow. The emergy of a flow can be expressed as:

*Em* = *En* × *UEV* (3)

where *Em* (seJ) refers to the emergy of a flow, *En* (j/g/$) refers to the available energy of a flow and *UEV* (seJ/(j/g/$)) refers to unit emergy value of the flow. The *UEV*s of different items are different, higher UEV means that more emergy have to be invested in a production of one unit of a flow, indicating its higher position in a hierarchy of energy transformations. 

In this paper, we defined ecological security as the security degree of human existence and development un-affected by ecological destruction and environmental pollution in production, living and health [14]. The studies of ecological security not only include environmental security but also focus on the sustainable development to promote the harmonious unity of economy, society and natural environment. The method of emergy analysis can measure and analyze the whole natural environment and human socio-economic system, which is suitable for evaluating ecological security and contributes to coordinating the ecological environment and economic development.

**Figure 2:**
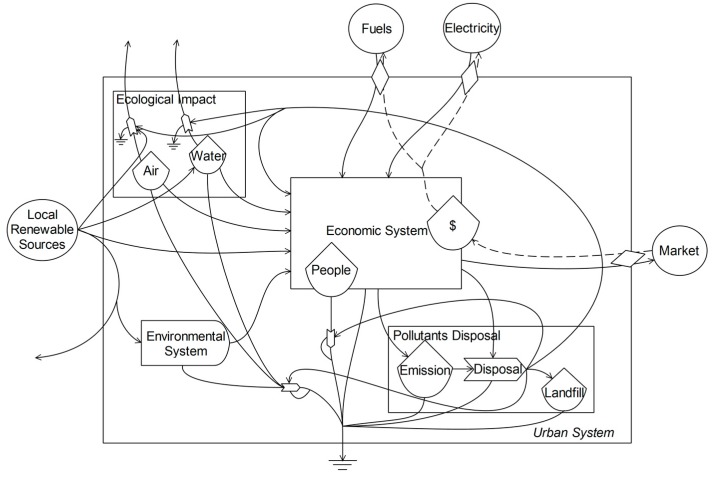
Diagram of the main emergy flows including emissions impact in urban system.

Most of the emergy research did not consider the emissions impact on ecological system at first. With the development of emergy analysis, the emissions impact gradually gained attentions and some scholars started to quantitatively calculate the emissions impact on ecological system and human health. Ulgiati, S. and Brown, M.T. calculated the environmental services required to dilute emissions from electricity production by means of emergy accounting methodology, which enabled us a way to quantify environmental services to drive the dilution process [37]. Ukidwe, N.U. and Bakshi, B.R. used disability adjusted life years (DALY) in the assessment framework of Eco-indicator 99 to analyze the emissions impact on human health [38]. Brown, M.T. and Ulgiati, S. calculated the emergy input for the restoration of ecosystem health [39]. Liu, G.Y. and Yang, Z.F. systematically analyzed the emissions impact on urban system by emergy analysis and presented the emergy calculation method of ecological impact [40]. Based on the previous research, we analyzed the ecological impact of emissions by emergy analysis, including ecological services needed to dissipate the emissions, human life losses, ecological losses, and land occupation, to comprehensively evaluate the ecological security. We have drawn an emergy diagram to show emergy flows of urban system including the emissions impact in Figure 2.

At present, the economic growth of China depends on large amounts of energy consumption, which made China one of the world’s largest energy users. In this paper, we comprehensively evaluated the ecological security from three aspects, economics, energy and environment. Considering that the energy consumption is the major source environmental pollution and closely related to the ecosystem, we regarded the energy consumption as the major component of imported inputs. Expect for the input sources, the flows of emissions also have an important influence on the whole system. The emissions from urban areas are discharged into natural environment after the treatment of pollutants, which have adverse effects on human life, ecological environment, ecological services and land resource. Human resource can be regarded as the local storage process of renewable resources, and every person has developed professional knowledge or capability by input resources. If human health and life are damaged by pollutants, the emergy of the input resources was lost. At the same time, pollutants can pollute the environment by acid rain and eutrophication, which will cause the losses of plants and animals. Due to the self-purification capacity of nature, the air and water of nature will provide extra ecological services, such as attenuation and degradation, to make pollutant concentration reach an acceptable concentration range for ecosystem. In addition, land occupation and degradation caused by landfill of solid waste will destroy land resource, too.

#### 3.2.2. Calculation of Ecosystem Emergy

The emergy of urban ecological system that was analyzed in this paper includes local renewable resources (R), local non-renewable resources (N), imported (non-renewable) inputs (F) and emissions impact (E). The local renewable resources (R) include solar radiation, wind, earth cycle, geo potential and chemical energy of rain. Local non-renewable resources (N) mainly include topsoil loss. Considering that the energy consumption is the major source of environmental pollution, the imported (non-renewable) inputs (F) considered in our study are fuels and electricity. In this paper, 12.0 × 10^24^ seJ/year was adopted as the planetary emergy baseline [41] and the UEV was adjusted accordingly. For the calculation procedure and UEV reference of local renewable resources (R), local non-renewable resources (N) and imported (non-renewable) inputs (F) see Appendix A (Table A1).

According to statistical data of environment protection bureau [25,26,27,28,29,30,31], the major emissions in Wuhan are waste water, waste gas and solid wastes and the main types of pollutant are SO_2_, NO_x_, COD, NH_3_-N, industrial dust, soot and hexavalent chromium. Based on the findings of Brown M.T. et al. [39] and LIU G.Y. et al. [40], the emergy of emissions impact (E) of Wuhan ecosystem was calculated, including emergy of (i) ecological services needed to dissipate the emissions (E_1_); (ii) human life losses caused by the emissions (E_2_); (iii) ecological losses due to the emissions (E_3_); and (iv) land occupation caused by the emissions (E_4_).

The emergy of ecological services needed to dissipate the emissions (E_1_) can be calculated by

(4) $$E_{1}=Em_{air}\times Em_{water}$$

where *Em_air_* represents the emergy that needed to dilute air pollution and *Em_water_* represents the emergy that needed to dilute water pollution.

(5) $$M_{air}=d\times \left(\frac{W}{c}\right)-M_{air}^{*}$$

(6) $$En_{air}=M_{air}\times \frac{v^{2}}{2}$$

(7) $$Em_{air}=En_{air}\times UEV_{air}$$

where *M_air_* represents the weight of air needed to dilute air pollution, *d* (1.3 kg/m^3^) represents the density of air, *W* represents the weight of pollutant in waste gas, *c* represents the standard of permitted environment level of pollutant, $M_{air}^{*}$ represents the weight of waste gas, *v* is average wind speed, *G* (4.94 J/g) represents the Gibbs free energy of water; *UEV_air_* represents unit emergy value of air.

(8) $$M_{water}=d\times \left(\frac{W}{c}\right)-M_{water}^{*}$$

(9) $$En_{water}=M_{water}\times G$$

(10) $$Em_{water}=En_{water}\times UEV_{water}$$

where *M_water_* represents the weight of water needed to dilute water pollution, *d* (1000 kg/m^3^) represents the density of water, *W* represents the weight of pollutant in waste water, *c* represents the standard of permitted environment level of pollutant, $M_{waer}^{*}$ represents the weight of waste water, *G* (4.94 J/g) represents the Gibbs free energy of water; *UEV_water_* represents unit emergy value of water.

The emergy of the human life losses caused by the emissions (E_2_) can be measured by

(11) $$E_{2}=\sum m_{i}\times DALY_{i}\times E_{m\;p.c.}$$

where *i* represents the types of pollutant, *m_i_* represents the weight of the chemical in emissions, *DALY_i_* is the influence factor in assessment framework of Eco-indicator 99 [42], and *E_m p.c._* is the emergy per capita in the study region, the total emergy equals to the sum of local renewable resources (R), local non-renewable resources (N) and imported (non-renewable) inputs (F).

The formula of emergy of the ecological losses due to the emissions (E_3_) is as follows:

(12) $$E_{3}=\sum m_{i}\times PDF_{i}\times E_{bio}$$

where *i* represents the types of pollutant, *m_i_* represents the weight of the chemical in emissions, *PDF_i_* is the proportion of potential extinction from assessment framework of Eco-indicator 99 [42], and *E_bio_* represents the emergy of unit biological resources.

The emergy of the land occupation caused by the emissions (E_4_) can be measured by total solid emissions and annual average emergy of unit land, the formula is:

(13) $$E_{4}=Area\times UEV_{land}$$

where *Area* represents the area of land occupied by solid waste, *UEV_land_* represents the unit emergy value of land. According to previous studies [43,44], 2.85 × 10^4^ t solid waste can take about 1 ha land and the annual average emergy of unit land is 7.98 × 10^4^ seJ/ha (converted to the new baseline [41]).

#### 3.2.3. Evaluating Indicators System Based on Emergy 

Resources are a crucial element of economic development and prosperity. However, consumption of resources will generate pollutants and endanger the natural environment. When emissions of pollutants exceed the critical value of environmental capacity, the environmental pollution will affect public health and threaten ecological security. The indicators system was established based on the relationship among economic growth, resource consumption and pollutant discharge to evaluate the ecological security status. The indicators are shown in Table 1.

The environment loading ratio indicates the pressure of non-renewable resources (N + F) and waste on the environment. The smaller the value of environment loading ratio is, the greater the extent of dependence on renewable resources will be. A natural ecosystem can entirely rely on the input of local renewable resources. And a higher value of environment loading ratio indicates more pressure on local environmental resources. Environmental potential is the proportion of local renewable resources to the total emergy of system, which reflects the potential capability of natural environment to support the whole system. And the ecological cost per GDP index was used to measure the environmental costs based on emergy. Heavy-polluting industries, underdeveloped technology and imperfect environmental policies will increase the ecological cost per GDP due to eco-environmental pollution. Emissions impact per energy consumption describes the eco-environmental pollution under unit energy consumption. It is mainly influenced by technology, energy structure and environmental policies.

#### 3.2.4. Emergy-Based Ecological Security Index

An emergy-based ecological security index (EESI) was established for analyzing the ecological security level of the study area [45]. The formula is as follows:

(14) (EESI)j=(GDP)i(GDP)j[PiPj]×[FiFj]×[EiEj]

where (EESI)*_j_* represents the emergy-based ecological security index in the *j* year, (*GDP*)*_j_* is Gross Domestic Product in the *j* year, *P_j_* is the registered population in the *j* year, *F_j_* is the imported inputs (energy consumption) in the *j* year, *E_j_* is the emergy of emissions impact in the *j* year, (*GDP*)*_i_*, and *P_i_*, *F_i_*, and *E_i_* indicate the corresponding indices in the reference year, respectively, which is 2006 in this paper.

EESI was introduced to describe the sustainability of social-economic development and ecological environment and reflect the regional ecological security. Compared with the reference year, the growth of EESI shows that economy, population, energy consumption and ecological environment are developing in the direction of harmony. A higher value of EESI reflects the improvement of ecological sustainability and security. EESI is applicable to ecological security assessment on the regional scale through comprehensive analysis of economy, energy and environment, which can help researchers discuss the variations of ecological security between different years.

### 3.3. Coupling Coordination Analysis 

The coupling coordination analysis was adopted in this paper to analyze the relationship between land use and ecological security for achieving coordinated development. The concept of coupling is used to describe the phenomenon of mutual-influence and interaction between systems [46]. The coordination is defined as the level of harmony and compatibility in the development of systems [47,48]. In order to quantitatively evaluate the coupling coordinated relationship, the coupling degree and coordination degree are introduced to study the interaction and influence between two systems—land use and ecological security, which can show the health status of the systems in the process of development.

The coupling degree is used to indicate the strength of interaction and mutual-influence between systems [49]. The coupling degree is calculated by

(15) $$O={\left[U\times L/{\left(\frac{U+L}{2}\right)}^{2}\right]}^{2}$$

where *O* refers to the coupling degree, *U* represents the index of land use change, and *L* represents the level of ecological security.

Then, the coordination degree was introduced to reflect the level of comprehensive coordinated development between systems. The coordination degree is calculated by

(16) $$D=\sqrt{O\times \left(\alpha U+\beta L\right)}$$

where *D* refers to the coordination degree, *U* represents the index of land use change, *L* represents the level of ecological security, and α and β. refer to weight coefficients and α + β = 1.

Because the land use and ecological security were at the same level of importance, it was set that α = β = 0.5 [46]. A higher value of coordination degree shows that the level of the coordination development is higher and the coupling relationship is more harmonious. Accordingly, the coupling coordination degree of land use change and ecological security were calculated to find the variation and tendency of coordinated development.

## 4. Results

### 4.1. Analysis of Land Use Change

**Figure 3:**
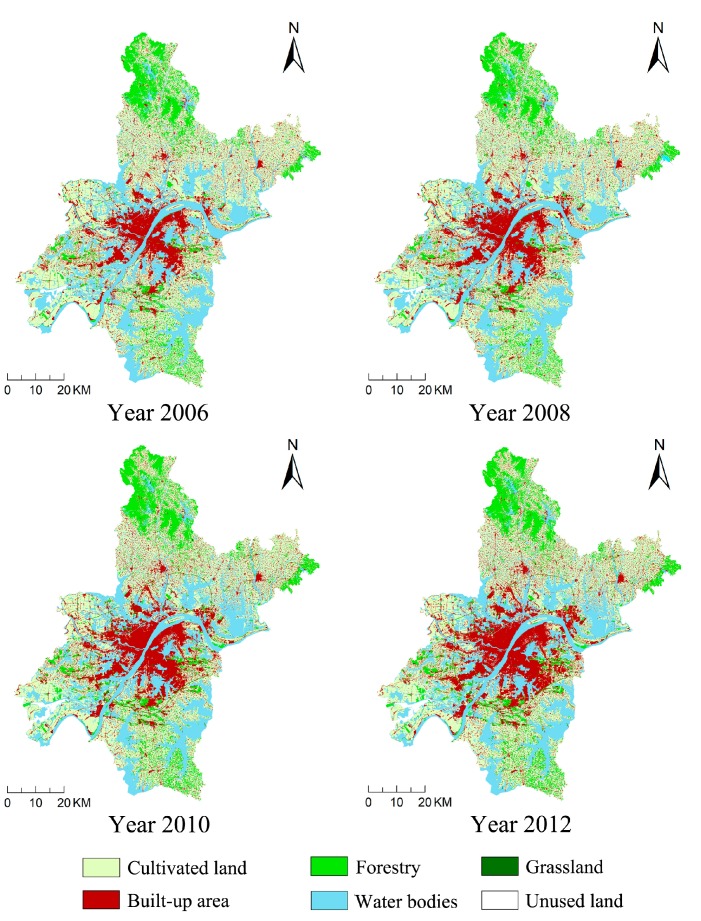
Maps of land use in the study region for the years of 2006, 2008, 2010 and 2012.

The status of land use in Wuhan, including land area and structure, was analyzed using the graphic editor function and the spatial analysis faculty of the Arc INFO platform. The land use data of 2006, 2008, 2010 and 2012 are shown in Figure 3.

**Figure 4:**
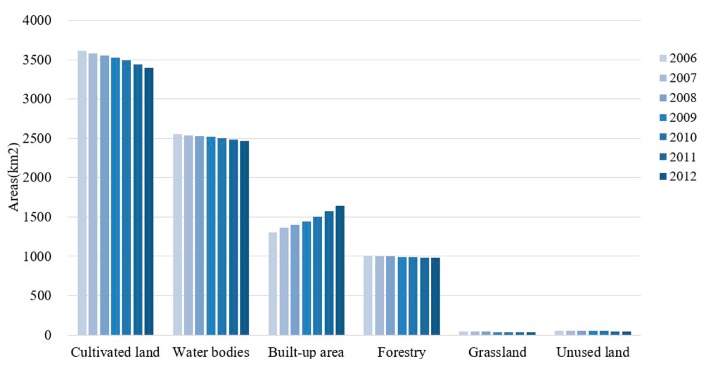
Structure of land use in Wuhan from 2006 to 2012.

The total land area of Wuhan is 8569.36 km^2,^ and the structure of land use is presented in Figure 4. Cultivated land, water bodies, built-up area and forestland were the main land use types in Wuhan city, which accounted for over 98.9% of the total area of Wuhan city from 2006 to 2012. According to the statistical results, the grassland and unused land took up less than 0.5% and 0.6% respectively from 2006 to 2012, so their effects on land use were negligible. Among the main land use types, the area used for construction kept expanding each year from 2006 by encroaching on cultivated land, water bodies and forestry, when its proportion increased from 15.23% in 2006 to 19.21% in 2012. Cultivated land, water bodies and forestry, which played an important role in the natural environment and ecological security, were all decreased during the study period. With the development of urbanization, more built-up land will be needed and the size of ecological land will continue to be reduced.

During the entire study period, cultivated land, water bodies and forestland all shrank while the built-up area increased (Table 2). From 2006 to 2007, built-up area increased the most, with a dynamic degree of 4.33%. And cultivated land was mainly converted into built-up area, which the dynamic degree was −1.04%, decreased faster than water bodies and forestry. From 2007 to 2009, the dynamic degree of built-up area dropped from 3.88% to 2.83%, with slackened growth. And the dynamic degree of forestry dropped from −0.43% to −0.50% showed that the degradation of forestry was aggravated and more forestry converted to built-up area. In this period, the change of cultivated land and water bodies showed that their pace of decline was slowed down than 2006 to 2007. From 2009 to 2012, the dynamic degree of built-up area increased from 3.78% to 4.62%, which was much higher than those of other land use types. Compared to the previous period, the change in built-up area between 2009 and 2012 was more intense. Meanwhile, the change of cultivated land, water bodies and forestry showed that the more and more ecological land was occupied by built-up area expansion. The occupation was mainly distributed around the built-up area (Figure 3).

The land use dynamic degree (Rd) and land use integrated dynamic degree (Rt) of each type of land presented in Table 2 and Table 3.

The integrated dynamic degree of land use gave an overall impression of intensity of land use type from 2006 to 2012 (Table 3). From 2011 to 2012, the integrated dynamic degree increased to 1.69%, indicating a more intense change in land use compared to the previous period. The variation tendency of the integrated dynamic degree of land use was basically consistent with the dynamic degree of built-up area, which illustrated that the urbanization and expansion of built-up area became the main driving force of land use change in Wuhan. The urbanization in Wuhan was a constantly changing process, which developed relatively fast during 2006 to 2007 and during 2011 to 2012. From 2006 to 2008, there was a stage of relatively rapid growth, with an annual growth rate of 3% to 4%. Then came the steady built-up area expansion between 2009 and 2011, with a growing rate of 2% to 3% per year. After 2011, it reverted to a rapid growing stage with the growth rate of 4.62% per year.

### 4.2. Assessment of Ecological Security Based on Emergy 

In order to evaluate ecological security in Wuhan, the data on materials, energy, and emissions in Wuhan from 2006 to 2012 were used to calculate the solar emergy of local renewable resources (R), local non-renewable resources (N), imported (non-renewable) inputs (F), and emissions impact (E). The solar emergy values of each item are shown in Table 4. For the calculation procedure and UEV reference of local renewable resources (R), local non-renewable resources (N) and imported (non-renewable) inputs (F) see Appendix A (Table A1).

According to the results of emergy accounting, the emergy flows of Wuhan in 2006–2012 were calculated, as are shown in Table 5.

In order to avoid double counting, the maximum value of wind, rain (geopotential), rain (chemical) and earth cycle was only taken into account. From 2006 to 2012, the total emergy flow of Wuhan increased for 45.83%, which showed that the emergy flux of ecological system in Wuhan had a remarkable development. At the same time, the emergy flow of imported inputs increased significantly to 1.91 × 10^23^ in 2012, which was 1.73 times of that in 2006. It showed that energy consumption of Wuhan was on a fast increase during this period. In addition, the emergy of ecological services needed to dissipate the emissions that increased for 38.84% from 2006 to 2012, showing that Wuhan needed more ecological services based on self-purification to eliminate the impact of pollutant emissions. The emergy of human life and ecological losses caused by the emissions both decreased to some degree. The reason is these emergy flows caused by SO_2_ and NO_x_, the main pollutants of human life and ecological losses, decreased in the same period (Table 4). The emergy of the land occupation caused by the emissions also decreased to 1.91 × 10^16^ in 2012 showed that the ecological impact of solid waste became smaller compared to the previous period. 

Table 6 presents the indicators for evaluating the ecological security of Wuhan. From 2006 to 2011, the environment loading ratio increased from 0.43 to 0.73, and then reduced to 0.54 in 2012. This showed that the pressure of non-renewable resources (N + F) and waste on environment was greater. The environmental potential decreased by 17.32% from 2006 to 2011 due to the significant growth of imported inputs, which increased for 62.81% in the same period from Table 5. The change of Ecological cost per GDP, had sharply declined by 73.26% from 2006 to 2012, showed that environmental cost in Wuhan was constantly reduced with the development of economy and urbanization. Emissions impact per energy consumption was on a decline from 0.05 in 2006 to 0.04 in 2012, which showed that the advances in technology, energy structure changes and environmental policies on pollution control and management were working, but not obvious.

**Figure 5:**
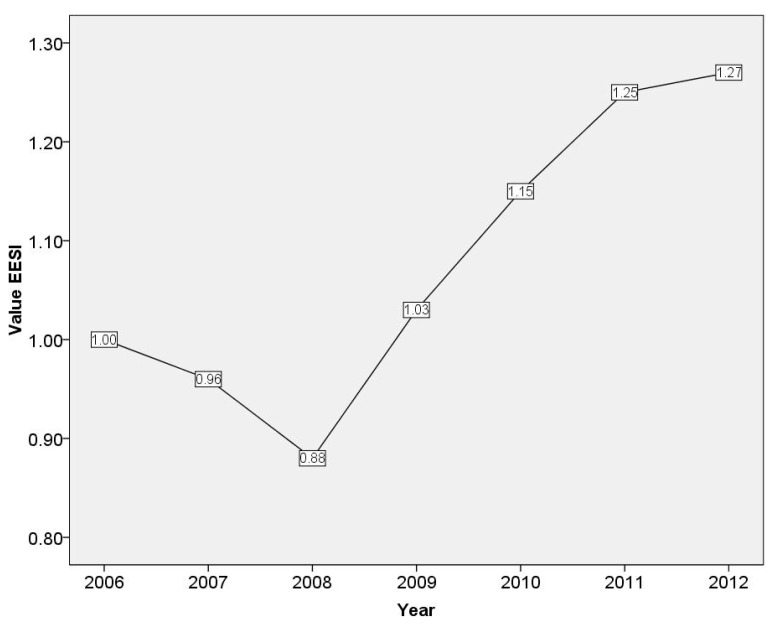
Variation curve of the emergy-based ecological security index for Wuhan.

As is described in Formula (14), the year of 2006 was the reference year in this paper, in which the emergy-based ecological security index (EESI) was 1.00. From Figure 5, it can be found that the variation trend of EESI presented a V-type change during the study period. The decline of EESI accelerated between 2006 and 2008. Then, the EESI increased to 1.28 in 2012, presenting a decrease-to-increase tendency. However, from 2011 to 2012, the growth rate of EESI slowed down considerably. Although the emissions impact in 2008 and 2012 were almost the same, as is shown in Table 5, the ecological cost per GDP of 2008 was much higher than that of 2012, which showed that the development of economy and natural environment in 2012 was more sustainable and harmonious than that in 2008. The variation tendency of EESI revealed that the overall level of ecological security in Wuhan rose slowly, although the level dropped in a certain degree during 2006–2008.

### 4.3. Coupling Coordination Analysis 

**Figure 6:**
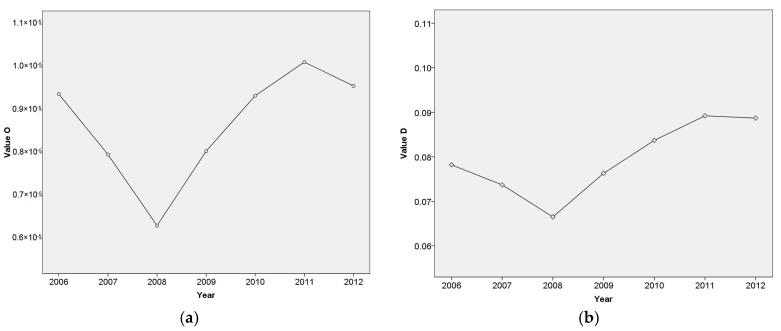
(**a**) Variation curve of coupling degree of built-up area and emergy-based ecological security index; (**b**) Variation curve of coupling coordination degree of built-up area and emergy-based ecological security index.

Considering that built-up area was the main source of pollution for the ecological environment and that the development of urbanization was the main driving force of land use change, the quantitative relationship between built-up area and emergy-based ecological security index (EESI) was studied using the coupling coordination analysis. The changing trends of the coupling coordinated relationship between built-up area and EESI is shown through the calculation results of coupling and coordination degree in Figure 6.

Figure 6 showed that the coupling coordination degree of built-up area and EESI rose in the undulation and the relationship between them tended to be more coordinated after 2008. Based on the variation characteristic, we divided the coupling coordinated relationship between built-up area and EESI into 3 stages: 2006 to 2008, 2008 to 2011 and 2011 to 2012. From 2006 to 2008, because the level of ecological security in Wuhan constantly dropped while built-up area expanded by about 4.11% per year, the coupling degree and coordination degree both decreased, which showed that the synchronization between built-up area and EESI gradually reduced and the development of them was uncoordinated. In this stage, the ecological security of Wuhan lagged behind the development of urbanization. From 2008 to 2011, the level of the coupling coordinated development of ecological security and built-up area showed a rising trend. Due to the EESI presented an increase tendency and the increasing rate of built-up area was controlled at 3.46% per year, which was slower than previous period, the development of EESI and built-up area was getting more harmonious and synchronous. From Figure 6a, it can be found that the interaction and mutual-influence between ecological security and built-up area were also strengthened. In this stage, the relationship of built-up area and ecological security evolved to be well coordinated, which revealed that the ecological security in Wuhan developed at the same pace with urbanization. From 2011 to 2012, the coupling and coordination degree of built-up area and EESI showed a downward trend again. The built-up area increased about 4.62% from 2011 to 2012, which was the fastest among all periods. This showed that the level of coupling coordinated development of ecological security and built-up area declined when built-up area expanded too rapidly.

## 5. Discussion

### 5.1. Driving Forces of Land Use Change in Wuhan City

Our study area, Wuhan city, from 2006 to 2012 experienced substantial land cover change, which led to rapid increase in built-up area, severe loss of cultivated land and water bodies. With the built-up area expansion speeding up, more and more cultivated land, water bodies and forestry were occupied for construction. Although both the transition of the natural environmental and social economic activities drive changes in land use, the effect of social economic activities is larger in the short term. The urbanization and expansion of built-up area were the main driving forces of land use change in Wuhan. Since 2006, driven by the reform and opening-up, Wuhan city has exploited its superior resources and privileged location to develop economy. Economic power and living standard of citizens have improved greatly, but, extensive resource utilization also brings ecological problems, including the sharply increasing area used for construction and the serious environmental pollution. Because of the economic crisis and holistic control over the construction land in 2008, the speed of urbanization in Wuhan slowed down and the land use dynamic degree of built-up area was 2.83% during 2008 to 2009. However, under the policies of expanding domestic demand and constructing Wuhan megalopolis after 2009, the speed of urbanization in Wuhan was accelerated and the land use dynamic degree of built-up area was 4.62% in 2012, which was highest during study period.

### 5.2. Main Factors Influencing Ecological Security Change in Wuhan City

The emergy analysis allow us to investigate and evaluate the whole natural environment and human socio-economic system by converting materials and energies to the same unit (seJ). From results of emergy analysis, we found that the main factors influencing ecological security are human activities and land use change with the following reasons. The environment loading ratio increased and the environmental potential decreased during 2006 to 2012 due to the growth of energy consumption and emissions impact. With the built-up area expansion and human activities growing intensely, more and more energy have to be consumed to meet the needs of daily life, construction, transportation and industrial manufacture, which also produced huge amounts of waste to the environment at the same time. When paying attention only to urbanization and economy, humans seriously threatened the ecological security with the model of inefficient and blind development. In order to prevent environmental pollution and degradation, a series of policies had been imposed by Wuhan government after 2008. Using the desulfurization equipment and improving production technology in industrial production to reduce the emission of SO_2_ and NO_x_. Promoting the upgrade of industrial structure and developing the third industry to strengthen pollution source control, reduce pollutant load and control sewerage total amount, such as shutting down energy-inefficient cement, steel and other heavy industries. Based on these efforts, the emergy losses caused by SO_2_, NO_x_, and Soot were decreased. During 2009 to 2012, the level of ecological security in Wuhan also has demonstrated an increased tendency, which reflected that the ecological environment was getting more sustainable and harmonious due to energy structure changes and emission reduction policies in the built-up area. Nevertheless, the energy consumption and other pollutants (e.g., NH_3_-N) still presented significant growth with urbanization, which may have negative effects on the level of ecological security in the future.

### 5.3. Implications of Land Use and Environmental Management According to Coupling Coordination Analysis

Land use change and ecological security both are essential to human well-being and sustainable development. The results of coupling coordination analysis showed that the relationship between land use change and ecological security tended to be more coordinated after 2008. During the period of 2009 to 2011, the increasing rate of built-up area was controlled at 3.46% per year, which was slower than that during 2006 to 2008 and 2011 to 2012. Furthermore, the emergy losses caused by SO_2_, NO_x_, and Soot were decreased from 2009 to 2011 since the policies of environmental protection, such as industrial structure change and emission reduction, were carried out. This revealed that the synchronous and coordinated development of land use change and ecological security can be achieved due to the reasonable expansion of built-up area and strict environmental protection. But, from 2011 to 2012, the coupling and coordination degree of built-up area and EESI showed a downward trend. In this period, the land use dynamic degree of built-up area was 4.62%, much higher than previous period. With the rapid urbanization, energy consumption and the other pollutants (e.g., NH_3_-N) increased significantly, which made the development of land use change and ecological security uncoordinated and disharmonious. Therefore, more decision-makings or polices should be proposed in Wuhan city. For land use change, the urbanization need to be controlled and limited with a reasonable speed, probably under 4% per year based on above. The policy of intensive land use should be implemented and the management of land transition should be strengthened, especially in construction land planning and supervision. For ecological security, it is necessary to emphasize energy-saving and emission reduction and exploit clean energy to change the structure of energy consumption. Moreover, more efforts are needed in pollution treatment to cut down the effects of environmental pollution.

### 5.4. Comparison with Previous Studies and Further Research Prospects

Previous studies has explored theoretical hypotheses, basic concepts and evaluation methods related to ecological security in China and investigated the relationship of land use change and ecological security [1,10,14,15]. Chen, H.S. et al. evaluated the ecological footprint and ecological capacity by using ecological footprint model to estimate the value of ecosystem service and ecological security [52]. But, the method of ecological footprint ignored the impacts of air and water pollution on ecological environment, which cannot accurately evaluate the level of ecological security. Han, B.L. et al. evaluated the ecological security in Beijing-Tianjin-Hebei metropolitan region based on fuzzy and entropy methods from the aspects of pressure, state and response [53]. Although they took the pollution into account in assessment, the fuzzy method cannot fully and quantitatively reflect the impacts of pollutants on environment and human health. Comparing with these methods, emergy analysis can calculate and analyze the emergy flows of whole ecological system, including the emissions impacts, to quantitatively evaluate the ecological security. The method of emergy analysis employed in this paper was more appropriate for the assessment of ecological security. Cen, X.T. et al. investigated the coupling relationship between intensive land use system and landscape ecological security system for urban sustainable development [54]. Their results also showed that the coupling coordinated model can provide a holistic view for identifying how to balance socio-economic and ecological environment under rapid urbanization.

However, there are still some problems that need to be solved in further research. Due to the limited annual statistical data, some pollutants, such as N_2_O, Arsenic, and Cyanide were not taken into account. And there might be a part of double counting when we calculating the emergy of emissions impact. Moreover, to achieve efficient use of land resources and sustainable ecological development, further research should also involve the macro analysis of ecological security and land use change on the temporal and spatial scales.

## 6. Conclusions

In this paper, the interaction between and coordinated development of land use change and ecological security were examined using the emergy analysis and coupling coordination analysis. Wuhan city was selected as the case study area due to its obvious land use change, ecologically representative terrain, and rapid economic growth. The conclusions of this research are summarized as follows.

The results of land use change analysis showed that the built-up area constantly increased from 2006 to 2012 while all the other land use types reduced in this period, reflecting that the development of urbanization was the main driving force of land use change in Wuhan. Due to the built-up area was the source of major pollutants, its scale should be under control and the economical and intensive land use should be promoted on the premise of the sustainable economic and social development.

According to the emergy analysis, the emergy-based indices were built to evaluate the ecological security of Wuhan city. From 2006 to 2011, the environment loading ratio increased from 0.43 to 0.73, and then reduced to 0.54 in 2012. The environmental potential decreased by 17.32% from 2006 to 2011. The change of Ecological cost per GDP, had sharply declined by 73.26% from 2006 to 2012. Emissions impact per energy consumption was on a decline from 0.05 in 2006 to 0.04 in 2012. From the results of EESI, it can be found that the level of ecological security in Wuhan showed an increasing trend as a whole after it dropped from 2006 to 2008. The variation tendency reflected that the ecological environment was getting more sustainable and harmonious. 

The variation curve of the coupling coordination degree of the built-up area and EESI revealed that the relationship between them tended to be more coordinated after 2008. Based on the variation characteristic, the coupling coordinated relationship between built-up area and EESI can be divided into 3 stages: 2006 to 2008, 2008 to 2011 and 2011 to 2012, which rose in the undulation. For achieving coordinated and sustainable development, the policy makers should spend more efforts on reasonable urbanization, intensive land use, and clean energy development, emission reduction.

To achieve efficient use of land resources and sustainable ecological development, further research should involve the macro analysis of ecological security and land use change on the temporal and spatial scales. Above all, this paper proposes that the coupling coordination degree model and emergy analysis be used for the integrated evaluation for land use change and ecological security to provide new ideas for land use management and environmental protection, and more references for decision making.

## Figures and Tables

**Table 1:** Emergy-based indicators considered in the study.

Index	Calculation	Unit
Environment loading ratio	(N + F + E)/R	-
Environmental potential	R/N + F + R	-
Ecological cost per GDP	(E_2_ + E_3_ + E_4_)/GDP	seJ/$
Emissions impact per energy consumption	E/F	-

R: local renewable resources; N: local non-renewable resources; F: imported (non-renewable) inputs; E: emissions impact; E_1_: emergy of ecological services needed to dissipate the emissions; E_2_: emergy of the human life losses caused by the emissions; E_3_: emergy of the ecological losses due to the emissions; E_4_: emergy of the land occupation caused by the emissions; E = E_1_ + E_2_ + E_3_ + E_4_.

**Table 2:** Land use dynamic degree (Rd) in Wuhan from 2006 to 2012.

Land Types/Period	2006–2007	2007–2008	2008–2009	2009–2010	2010–2011	2011–2012
Cultivated land	−1.04%	−0.65%	−0.78%	−0.98%	−1.31%	−1.45%
Water bodies	−0.58%	−0.37%	−0.50%	−0.56%	−0.77%	−0.67%
Built-up area	4.33%	3.88%	2.83%	3.78%	3.77%	4.62%
Forestry	−0.31%	−0.43%	−0.50%	−0.43%	−0.41%	−0.47%

**Table 3:** Land use integrated dynamic degree (Rt) in Wuhan from 2006 to 2012.

Period	2006–2007	2007–2008	2008–2009	2009–2010	2010–2011	2011–2012
Rt	1.32%	0.89%	1.10%	1.27%	1.66%	1.69%

**Table 4:** Emergy synthesis for the regional ecosystem of Wuhan from 2006 to 2012.

Note	Item	Unit	UEV (seJ/Unit)	Source *	Emergy (seJ/year)						
					2006	2007	2008	2009	2010	2011	2012
Local Renewable resources (R)											
1	Sunlight	J/year	1	[50]	4.84 × 10^19^	4.84 × 10^19^	4.84 × 10^19^	4.84 × 10^19^	4.84 × 10^19^	4.84 × 10^19^	4.84 × 10^19^
2	Wind	J/year	1.91 × 10^3^	[50]	3.23 × 10^22^	3.23 × 10^22^	3.23 × 10^22^	3.23 × 10^22^	3.23 × 10^22^	3.23 × 10^22^	3.23 × 10^22^
3	Rain (geopotential)	J/year	1.32 × 10^4^	[50]	2.71 × 10^23^	3.16 × 10^23^	3.28 × 10^23^	3.00 × 10^23^	3.46 × 10^23^	2.55 × 10^23^	3.66 × 10^23^
4	Rain (chemical)	J/year	2.32 × 10^4^	[50]	1.03 × 10^22^	1.20 × 10^22^	1.24 × 10^22^	1.14 × 10^22^	1.31 × 10^22^	9.69 × 10^21^	1.39 × 10^22^
5	Geothermal heat	J/year	5.38 × 10^4^	[50]	5.44 × 10^20^	5.44 × 10^20^	5.44 × 10^20^	5.44 × 10^20^	5.44 × 10^20^	5.44 × 10^20^	5.44 × 10^20^
Local Non-renewable resources (N)											
6	Topsoil loss	J/year	9.35 × 10^4^	[50]	1.08 × 10^21^	1.06 × 10^21^	1.06 × 10^21^	1.05 × 10^21^	1.04 × 10^21^	1.03 × 10^21^	1.01 × 10^21^
Imported (non-renewable) inputs (F)											
7	Coal	J/year	5.08 × 10^4^	[50]	3.33 × 10^21^	3.94 × 10^21^	3.85 × 10^21^	1.82 × 10^22^	2.12 × 10^22^	1.82 × 10^22^	2.13 × 10^22^
8	Coke	J/year	8.36 × 10^4^	[51]	1.08 × 10^22^	1.04 × 10^22^	1.24 × 10^22^	1.19 × 10^22^	1.58 × 10^22^	1.64 × 10^22^	1.54 × 10^22^
9	Crude Oil	J/year	6.90 × 10^4^	[51]	1.17 × 10^22^	1.24 × 10^22^	1.15 × 10^22^	1.31 × 10^22^	1.44 × 10^22^	1.46 × 10^22^	1.25 × 10^22^
10	Gasoline	J/year	7.98 × 10^4^	[51]	2.36 × 10^22^	2.53 × 10^22^	3.22 × 10^22^	3.23 × 10^22^	3.36 × 10^22^	3.62 × 10^22^	4.12 × 10^22^
11	Kerosene	J/year	8.36 × 10^4^	[51]	4.95 × 10^22^	5.46 × 10^22^	6.64 × 10^22^	7.28 × 10^22^	7.51 × 10^22^	7.55 × 10^22^	8.09 × 10^22^
12	Liquefied petroleum gas	J/year	8.44 × 10^4^	[51]	1.11 × 10^19^	1.56 × 10^19^	1.63 × 10^19^	1.76 × 10^19^	2.87 × 10^19^	2.05 × 10^19^	1.43 × 10^19^
13	Electricity	J/year	1.32 × 10^5^	[50]	1.10 × 10^22^	1.36 × 10^22^	1.36 × 10^22^	1.48 × 10^22^	1.68 × 10^22^	1.83 × 10^22^	1.92 × 10^22^
Emissions Impact (E)											
Ecological services needed to dissipate the emissions (E_1_)											
14	SO_2_	J/year	**	-	7.75 × 10^19^	7.43 × 10^19^	6.89 × 10^19^	6.66 × 10^18^	5.15 × 10^19^	6.02 × 10^19^	5.87 × 10^19^
15	Industrial dust	J/year	**	-	1.94 × 10^18^	1.23 × 10^18^	1.18 × 10^18^	1.14 × 10^18^	1.19 × 10^18^	2.47 × 10^18^	2.31 × 10^18^
16	Soot	J/year	**	-	3.31 × 10^19^	3.11 × 10^19^	2.82 × 10^19^	2.36 × 10^19^	1.01 × 10^19^	2.10 × 10^19^	1.96 × 10^19^
17	NO_x_	J/year	**	-	4.68 × 10^19^	4.49 × 10^19^	4.17 × 10^19^	4.03 × 10^18^	3.11 × 10^19^	3.74 × 10^19^	3.46 × 10^19^
18	COD	J/year	**	-	2.37 × 10^21^	2.27 × 10^21^	2.20 × 10^21^	2.16 × 10^21^	2.10 × 10^21^	2.44 × 10^21^	2.31 × 10^21^
19	NH_3_-N	J/year	**	-	2.63 × 10^21^	3.47 × 10^21^	4.79 × 10^21^	3.82 × 10^21^	3.64 × 10^21^	3.89 × 10^21^	4.74 × 10^21^
Emergy of the human life losses caused by the emissions (E_2_)											
20	SO_2_	J/year	**	-	6.51 × 10^19^	6.25 × 10^19^	5.79 × 10^19^	5.60 × 10^18^	4.33 × 10^19^	5.06 × 10^19^	4.94 × 10^19^
21	NO_x_	J/year	**	-	1.60 × 10^20^	1.53 × 10^20^	1.42 × 10^20^	1.37 × 10^19^	1.06 × 10^20^	1.27 × 10^20^	1.18 × 10^20^
22	Industrial dust	J/year	**	-	4.50 × 10^19^	2.86 × 10^19^	2.73 × 10^19^	2.63 × 10^19^	2.76 × 10^19^	5.73 × 10^19^	5.34 × 10^19^
23	Hexavalent chromium	J/year	**	-	2.35 × 10^17^	2.06 × 10^17^	3.53 × 10^17^	7.64 × 10^17^	1.41 × 10^18^	9.99 × 10^17^	8.23 × 10^17^
Emergy of the ecological losses due to the emissions (E_3_)											
24	SO_2_	J/year	**	-	1.10 × 10^19^	1.06 × 10^19^	9.82 × 10^18^	9.49 × 10^17^	7.33 × 10^18^	8.58 × 10^18^	8.36 × 10^18^
25	NO_x_	J/year	**	-	9.15 × 10^19^	8.77 × 10^19^	8.14 × 10^19^	7.87 × 10^19^	6.08 × 10^19^	7.30 × 10^19^	6.76 × 10^19^
Emergy of the land occupation caused by the emissions (E_4_)											
26	Solid wastes	J/year	**	-	2.84 × 10^16^	1.82 × 10^16^	2.45 × 10^16^	3.09 × 10^16^	5.27 × 10^15^	9.35 × 10^15^	1.91 × 10^16^

*: the UEV was converted to the new baseline: 12.0 × 10^24^ seJ/year [41]. **: the emergy of emissions impact were calculated according to formulae (4)–(13).

**Table 5:** Emergy flows calculated for Wuhan.

Emergy Flows	Value (seJ/year)						
	2006	2007	2008	2009	2010	2011	2012
R	2.71 × 10^23^	3.16 × 10^23^	3.28 × 10^23^	3.00 × 10^23^	3.46 × 10^23^	2.55 × 10^23^	3.66 × 10^23^
N	1.08 × 10^21^	1.06 × 10^21^	1.06 × 10^21^	1.05 × 10^21^	1.04 × 10^21^	1.03 × 10^21^	1.01 × 10^21^
F	1.10 × 10^23^	1.20 × 10^23^	1.40 × 10^23^	1.63 × 10^23^	1.77 × 10^23^	1.79 × 10^23^	1.91 × 10^23^
E_1_	5.16 × 10^21^	5.89 × 10^21^	7.13 × 10^21^	6.01 × 10^21^	5.84 × 10^21^	6.45 × 10^21^	7.16 × 10^21^
E_2_	2.70 × 10^20^	2.44 × 10^20^	2.28 × 10^20^	4.64 × 10^19^	1.78 × 10^20^	2.36 × 10^20^	2.22 × 10^20^
E_3_	1.03 × 10^20^	9.83 × 10^19^	9.12 × 10^19^	7.96 × 10^19^	6.81 × 10^19^	8.16 × 10^19^	7.60 × 10^19^
E_4_	2.84 × 10^16^	1.82 × 10^16^	2.45 × 10^16^	3.09 × 10^16^	5.27 × 10^15^	9.35 × 10^15^	1.91 × 10^16^
E	5.53 × 10^21^	6.23 × 10^21^	7.45 × 10^21^	6.14 × 10^21^	6.08 × 10^21^	6.77 × 10^21^	7.46 × 10^21^
U	3.88 × 10^23^	4.44 × 10^23^	4.76 × 10^23^	4.70 × 10^23^	5.30 × 10^23^	4.42 × 10^23^	5.65 × 10^23^

U: the total emergy flow, U = R + N + F + E.

**Table 6:** Emergy-based indicators calculated for Wuhan.

Index	Value						
	2006	2007	2008	2009	2010	2011	2012
Environment loading ratio	0.43	0.40	0.45	0.57	0.53	0.73	0.54
Environmental potential	0.71	0.72	0.70	0.65	0.66	0.59	0.66
Ecological cost per GDP (seJ/$)	9.45 × 10^9^	7.26 × 10^9^	5.27 × 10^9^	1.86 × 10^9^	3.01 × 10^9^	3.20 × 10^9^	2.53 × 10^9^
Emissions impact per energy consumption	0.05	0.05	0.05	0.04	0.03	0.04	0.04

## Appendix A

**Table A1:** Calculation procedure and references for unit emergy value (UEV).

Item		Value	Unit	Reference
1	Sunlight			
	Area	=8.57 × 10^9^	m^2^	
	Sun radiation	=5.65 × 10^9^	J/(m^2^year)	[32]
	Energy	=(area) × (sun radiation)	J/year	
	UEV	=1	seJ/year	[50]
2	Wind			
	Area	=8.57 × 10^9^	m^2^	
	Air density	=1.23	kg/m^3^	
	Eddy diffusivity	=12.95	m^3^/s	[32]
	Wind velocity gradient	=3.93 × 10^−3^	m/s/m^2^	[32]
	Time frame	=3.15 × 10^7^	s/year	
	Energy	=(area) × (air density) × (eddy diffusivity) × (wind velocity gradient)^2^ × (time frame) × 1000	J/year	
	UEV	=1.91 × 10^3^	seJ/J	[50], converted to the new baseline
3	Rain (geopotential)			
	Area	=8.57 × 10^9^	m^2^	
	Average elevation	=23.3	m	[32]
	Annual rainfall		m/year	[32]
	Acceleration of gravity	=9.8	m/s^2^	
	Water density	=1000	kg/m^3^	
	Energy	=(area) × (average elevation) × (annual rainfall) × (acceleration of gravity) × (water density)	J/year	
	UEV	=1.32 × 10^4^	seJ/J	[50], converted to the new baseline
4	Rain (chemical)			
	Area	=8.57 × 10^9^	m^2^	
	Annual rainfall		m/year	[32]
	Gibbs energy of rain	=4.94	J/g	
	Water density	=1000	kg/m^3^	
	Energy	=(area) × (annual rainfall) × (gibbs energy of rain) × (water density)	J/year	
	UEV	=2.32 × 10^4^	seJ/J	[50], converted to the new baseline
5	Geothermal heat			
	Area	=8.57 × 10^9^	m^2^	
	Heat flux	=1.45 × 10^6^	J/m^2^/year	[32]
	Energy	=(area) × (heat flux)	J/year	
	UEV	=5.38 × 10^4^	seJ/J	[50], converted to the new baseline
6	Top soil			
	Cultivated area		m^2^	
	Erosion rate	=250	g/m^2^/year	[25,26,27,28,29,30,31]
	Organic matter in top soil	=56.4	%	
	Organic matter energy	=2.26 × 10^4^	J/g	
	Energy	=(cultivated area) × (erosion rate) × (organic matter in top soil) × (organic matter energy)	J/year	
	UEV	=9.35 × 10^4^	seJ/J	[50], converted to the new baseline
7	Coal			
	Coal consumption		t/year	[18,19,20,21,22,23,24]
	Energy content	=2.09 × 10^10^	J/t	
	Energy	=(coal consumption) × (en. content)	J/year	
	UEV	=5.08 × 10^4^	seJ/J	[50], converted to the new baseline
8	Coke			
	Coke consumption		t/year	[18,19,20,21,22,23,24]
	Energy content	=3.18 × 10^10^	J/t	
	Energy	=(coke consumption) × (en. content)	J/year	
	UEV	=8.36 × 10^4^	seJ/year	[51], converted to the new baseline
9	Crude oil			
	Crude oil consumption		t/year	[18,19,20,21,22,23,24]
	Energy content	=4.18 × 10^10^	J/t	
	Energy	=(crude oil consumption) × (en. content)	J/year	
	UEV	=6.90 × 10^4^	seJ/J	[51], converted to the new baseline
10	Gasoline			
	Gasoline consumption		t/year	[18,19,20,21,22,23,24]
	Energy content	=4.61 × 10^10^	J/t	
	Energy	=(gasoline consumption) × (en.content)	J/year	
	UEV	=7.98 × 10^4^	seJ/J	[51], converted to the new baseline
11	Kerosene			
	Kerosene consumption		t/year	[18,19,20,21,22,23,24]
	Energy content	=4.35 × 10^10^	J/t	
	Energy	=(kerosene consumption) × (en.content)	J/year	
	UEV	=8.36 × 10^4^	seJ/J	[51], converted to the new baseline
12	Liquefied petroleum gas			
	Liquefied petroleum gas consumption		m^3^/year	[18,19,20,21,22,23,24]
	Energy content	=2.93 × 10^10^	J/m^3^	
	Energy	=(liquefied petroleum gas consumption) × (en. content)	J/year	
	UEV	=8.44 × 10^4^	seJ/J	[51], converted to the new baseline
13	Electricity			
	Electricity consumption		kWh/year	[18,19,20,21,22,23,24]
	Energy content	=3.60 × 10^6^	J/kWh	
	Energy	=(electricity consumption) × (en. content)	J/year	
	UEV	=1.32 × 10^5^	seJ/J	[50], converted to the new baseline
